# Physiological and Metabolic Changes Induced by Fullerene C_60_ Derivatives in Zinc-Stressed Cucumber

**DOI:** 10.3390/plants15020254

**Published:** 2026-01-14

**Authors:** Nikolai Bityutskii, Kirill Yakkonen, Roman Puzanskiy, Allexey Shavarda, Konstantin Semenov, Marina Nadporozhskaya

**Affiliations:** 1 Department of Agricultural Chemistry, Saint Petersburg State University, 7/9 Universitetskaya nab., Saint Petersburg 199034, Russia; k.yakkonen@spbu.ru; 2 Department of Analytical Phytochemistry, Komarov Botanical Institute, Russian Academy of Sciences, St. Professora Popova, 2, Saint Petersburg 197022, Russia; puzansky@yandex.ru (R.P.); stachyopsis@gmail.com (A.S.); 3 Center for Molecular and Cell Technologies, Saint Petersburg State University, Saint Petersburg 199034, Russia; 4 Department of General and Bioorganic Chemistry, First Pavlov State Medical University, 6-8 L’va Tolstogo Ulitsa, Saint Petersburg 197022, Russia; semenov1986@yandex.ru

**Keywords:** cucumber, zinc-toxicity, fullerene derivatives, metabolomics, alleviation

## Abstract

Zinc (Zn) in excess is very toxic for plants and can limit agriculture. Carbon-based engineered nanomaterials with high electron mobility and electron-accepting capability may be essential for mitigating heavy metal stress. In the present study, the protective role of some fullerene C_60_ derivatives (fullerenol [C_60_(OH)_22–24_] and the arginine C_60_ [C_60_(C_6_H_13_N_4_O_2_)_8_H_8_]) were tested for the first time against Zn toxicity in *Cucumis sativus* L. (cucumber). Plants were grown hydroponically at three concentrations of fullerenes (0, 2, and 10 mg L^−1^) without or with 40 µM Zn for 17 days. Plant growth, leaf chlorosis, and nutritional imbalances in combination with a metabolomics approach were analyzed. The Zn-treated plants show chlorotic leaves, the retarded growth of shoots (−20%), and roots (−49%) and nutrient imbalance. Addition of fullerene C_60_ derivatives suppressed loss in the dry biomass of leaves (15%) and roots (40%; fullerenol only) induced by high Zn. However, they did not alter leaf chlorophyll, shoot dry biomass, and elemental composition, including leaf Zn. Moreover, the Zn of xylem sup from roots remained unchanged by fullerenes. In an adsorption experiment, the amounts of Zn adsorbed by tested C_60_ were below the detection limits. The addition of C_60_ derivatives slightly changed the metabolite profiling in stressed plants. Nevertheless, in fullerene-treated plants, the abundance of some Zn-responsible metabolites tended to be altered in the opposite direction as compared with the metabolic responses to excessive Zn alone. There were several up-regulated metabolites protecting plants under oxidative stress. We speculate that fullerene C_60_ derivatives have the ability to increase antioxidant non-enzyme activity at least, improving some growth parameters. However, fullerenes did not reduce Zn transport from the root to the shoots. We concluded that the low capacity of these compounds to buffer Zn in the root zone might limit the efficiency of fullerene derivatives against Zn toxicity. Our results provide new evidence for the crucial role of Zn–fullerene interactions in the amelioration of Zn toxicity in plants.

## 1. Introduction

Zinc (Zn) is a vital element (essential micronutrient) for higher plants, which plays structural and/or catalytic functions in several physiological processes such as protein synthesis, cell division, cell expansion, gene expression, metabolism of nucleic acids, carbohydrates, lipids, etc. [1,2,3]. However, based on Zn density (>5 g/cm^3^), this element has been categorized as a heavy metal (HM), which can be harmful for the plants as well as for all other living organisms at elevated concentrations [3,4,5,6]. The general symptoms of Zn toxicity in plants are growth inhibition, leaf chlorosis and necrosis, nutrient imbalance, oxidative burst, and a reduced photosynthetic rate and water uptake [7,8,9]. Structural and functional abnormalities expressed by excessive Zn cause a hindrance to global agricultural productivity and the high quality of crop production [3]. Moreover, high Zn concentrations in tissues of crop plants increase the potential risk of contamination in plant–human food chains [3]. The main sources of Zn in soils are weathering of parent materials and anthropogenic activity [10,11,12]. In agricultural soils, additional sources of Zn input are phosphate fertilizers, manure, fungicides, and limestone [13].

Nanotechnology has greater potential for increasing crop protection. The high reactivity of engineered nanomaterials (ENMs) caused by a small size and large area offer new approaches for using ENMs to ameliorate HM toxicity in plants [14]. For example, titanium dioxide ENMs have been successfully used for the protection of plants against the toxicity of copper (Cu) and lead (Pb) [15]. After discovering carbon-based NPs, the application of nanomaterials rapidly increased. Fullerenes are carbon-based ENMs that have potential application in plant sciences and agriculture for increasing crop productivity and protection [14,16,17]. Fullerene C_60_ is a molecule made up of sixty carbon atoms that assemble in a quasi-spherical geometry [18]. Native fullerene is insoluble in water, which may prevent the application of these nanomaterials in biology. However, to date, many water-soluble fullerene derivatives have been synthesized that retain the properties of native fullerene, which afford them wide biological applications [19,20,21].

The unique ability of fullerenes to scavenge all forms of reactive oxygen species (ROS) produced within plants can help plants overcome various abiotic stresses [19]. Many authors have been reported that polyhydroxy fullerenes (fullerenols) can protect crops against oxidative stress induced by different stressful environments. Indeed, fullerenol enhanced the root elongation of *Hordeum vulgare* stressed by UV-B radiation, salt, and an excess of salicylic acid [22]. Alleviation of drought impact was found in sugar beets and *Arabidopsis* as well as Fe chlorosis in cucumber [23,24,25]. Recently, the role of fullerenols in the protection of crops against oxidative stress has been reviewed [19]. Although environmental heavy metal pollution is widespread, little is known about the effectiveness of fullerenes against HM toxicity in plants. Recently, several works have been published on the study of the protective role of fullerenes in excess of HMs such as copper [26,27,28] and cobalt [29]. However, the mechanism underlying the effects of fullerenes on plants growing under high concentrations of HMs remain unclear. Furthermore, little information is available on the effects of fullerenes on plants subjected to high Zn.

This study planned to investigate whether water-soluble derivatives of fullerene C_60_ (fullerenol and arginine derivatives) show potential to reduce Zn toxicity in cucumber plants grown hydroponically. Several physiological indicators were analyzed combined with a metabolomics approach to evaluate Zn tolerance in fullerene-treated plants. Here, the fullerene treatments and experimental design were generally the same as in our previous work, where we studied the protective properties of these fullerenes against excess copper (Cu). This approach allows us to discuss the specifics of the action of fullerene derivatives under stress conditions induced by different HMs such as Zn and Cu.

This study aimed, for the first time, to assess the impact of two functionalized fullerene C_60_ derivatives on the responses to Zn stress in cucumber plants. It was hypothesized that these fullerenes might be involved in the alleviation of Zn toxicity, but protective efficiency would be dependent on the Zn-sorption ability of these C_60_ compounds.

## 2. Results

### 2.1. Physiological Responses to Zn Excess and Fullerene Treatments

**Figure 1:**
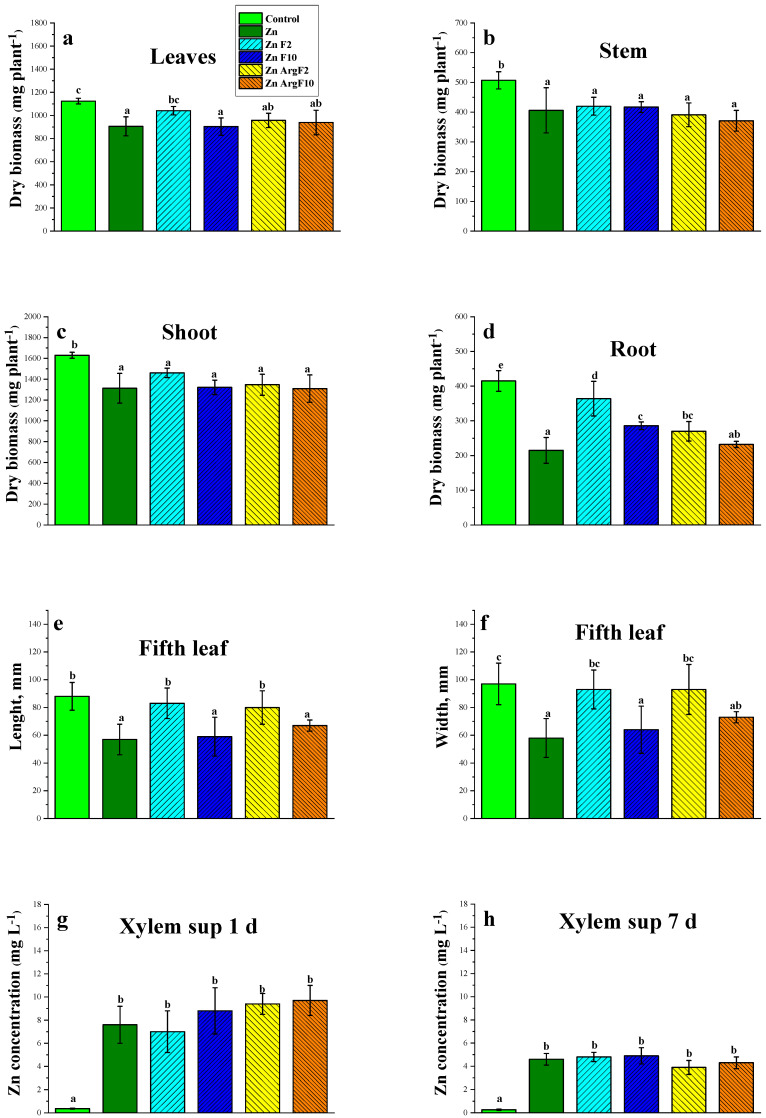
The effect of excess Zn on leaf (**a**), stem (**b**), shoot (**c**) and root (**d**) biomass, blade sizes of fifth (**e**,**f**) leaves, as well as on xylem sup Zn concentration (**g**,**h**) of cucumber plants cultivated in a nutrient solution with or without the supply of 2 (F2) and 10 (F10) mg L^−1^ fullerenol or 2 (ArgF2) and 10 (ArgF10) mg L^−1^ arginine C_60_ for 1 d (**g**), 7 d (**h**), and 17 d (**a**–**f**). Data represented as mean ± SD (*n* = 4). Significant differences between treatments (*p* < 0.05) are indicated by different letters.

When cucumber plants were grown in a nutrient solution with excessive Zn (40 µM), leaves began to develop chlorotic symptoms after a short time (3–4 d), resulting in morphological differences from the control plants. At harvest (after 17 d), the Zn-suffering plants showed retarded growth of aboveground (−20%) and especially of underground (−49%) parts in relation to the control plants (Figure 1a–d). The leaf blades of non-stressed plants were longer and wider (+35–40%) than that of plants subjected to Zn stress (Figure 1e,f).

The fullerene C_60_ adducts affected plants in a dose-dependent manner. Although there were no significant differences in leaf Chl between fullerene-treated and untreated +Zn plants, the +F2 and +ArgF2 plants exhibited larger (+45–60%) leaf (L5) sizes than the +Zn plants (Figure 1e,f). Moreover, at the end of the experiment, these fullerene C_60_ adducts increased the dry biomass of leaves and roots of Zn-stressed plants, being more pronounced in the fullerenol treatments at the lowest concentration of 2 mg L^−1^ (Figure 1).

### 2.2. Elemental Composition of Plants

As expected, excessive Zn led to increased content of the element in both xylem sup directed from roots to shoots and leaves of cucumber (Figure 1 and Table 1 and Table A1). Even after 1 d exposure, the Zn contents in xylem sup and leaves increased by 15- and 4-fold, respectively, as compared to the control plants. In leaves, enhanced Zn concentration lowered the content of the nutrients, such as Fe, B, P, K, Ca, and S, indicating imbalanced macro- and micronutrient compositions in the leaves and roots of Zn-stressed cucumber (Table 1). Neither fullerenol nor arginine fullerene C_60_ significantly changed the leaf and xylem sup Zn during the experiments (Figure 1 and Table 1).

### 2.3. General Characterization of Metabolome

**Figure 2:**
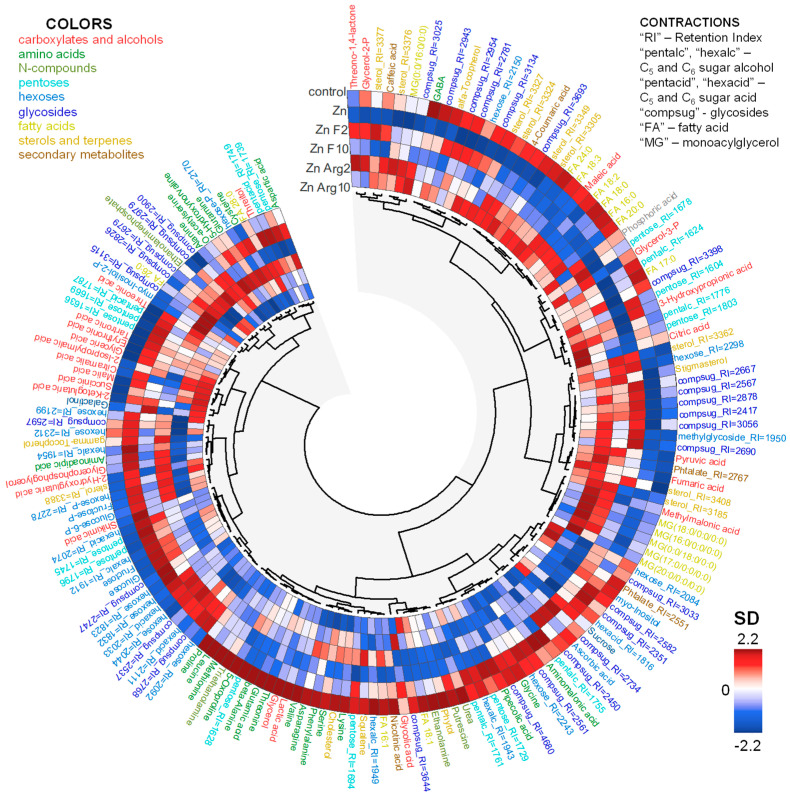
Heatmap of the mean metabolite content in the leaves of untreated control and Zn-stressed plants of cucumber cultivated with or without the supply of 2 (F2) and 10 (F10) mg L^−1^ fullerenol or 2 (ArgF2) and 10 (ArgF10) mg L^−1^ arginine C_60_ for 17 d. Data normalized per sample median, log-transformed, and standardized. Metabolites are clustered by Pearson correlation and Ward method.

The obtained metabolite profiles included approximately 320 compounds, of which 92 were identified, while the remaining 80 chemical classes were only annotated (Figure 2). The largest group was sugars, which included pentoses (17), hexoses (28), and complex sugars such as disaccharides and other various glycosides (32). Furthermore, 23 different amino acids, approximately two dozen carboxylic acids, 17 fatty acids and their derivatives, one and a half dozen sterols and terpenes, and several secondary compounds were identified.

### 2.4. Metabolic Responses to Excessive Zn and Fullerenol

**Figure 3:**
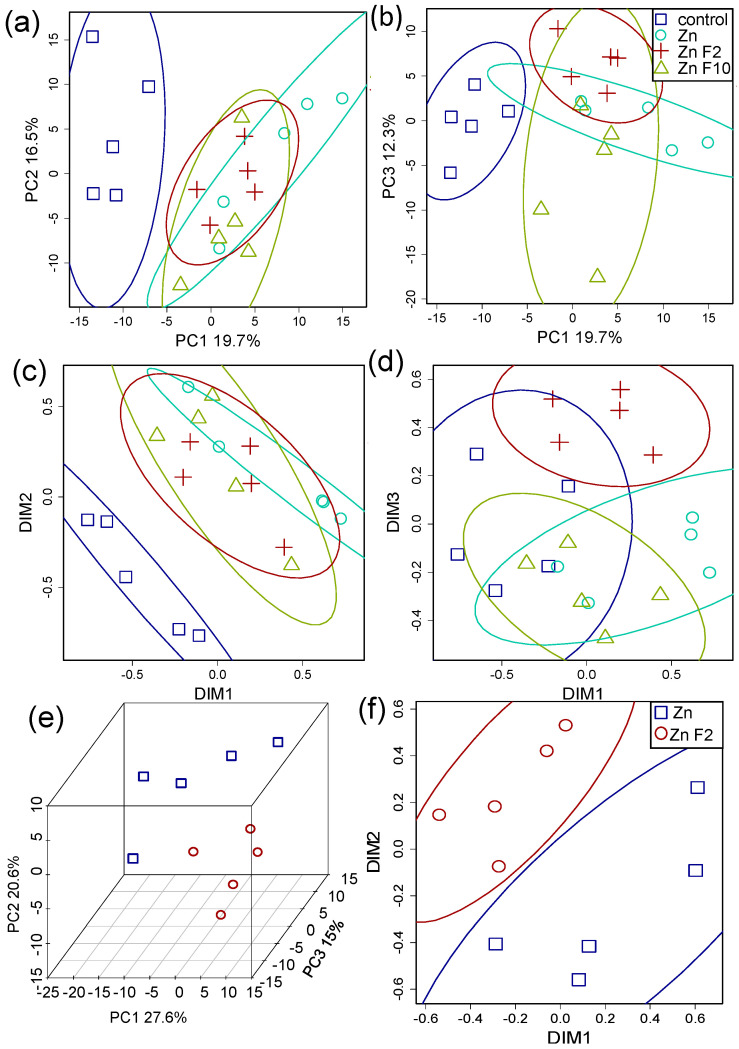
Metabolomic effects of Zn and fullerenol. The representation of metabolite profiles in the low-dimensional space. Score plots (**a**,**b**) obtained by principal component analysis (PCA). B. Scattering in the coordinates (**c**,**d**) extracted by multidimensional scaling (MDS) using 1 rho as a distance measure, where rho is Spearman’s correlation coefficient. Score plots (**e**) obtained by PCA and MDS derived dimensions (**f**) for Zn and ZnF variants. Dots correspond to the metabolite profiles, % is the percentage of variance related to the principal component (PC), and ellipses are 90% CI.

In order to facilitate the visualization of the similarity of metabolite profiles between each other, we represented them in a lower-dimensional space. A principal component analysis (PCA) was applied. The PCA score plot (Figure 3a,b) revealed that the metabolite profiles of control plants were clearly distinguishable from those observed in the Zn-treated plants along PC1, which explained 19.7% of the total variance.

The addition of fullerenol (Zn + F) had a weak effect on the metabolic profiles of Zn-treated plants (Zn). There was only a shift in the Zn + F samples along PC3 (12.3%). A similar pattern is obtained when dimensionality is reduced by MDS (multidimensional scaling) using the Spearman correlation as a similarity measure (Figure 3c,d). Separate consideration of the +Zn and the +F2/F10 variants revealed weakly pronounced differences between the +Zn and + F2 (Figure 3e,f). Thus, the addition of fullerenols results in only a weakly expressed metabolic phenotype.

**Figure 4:**
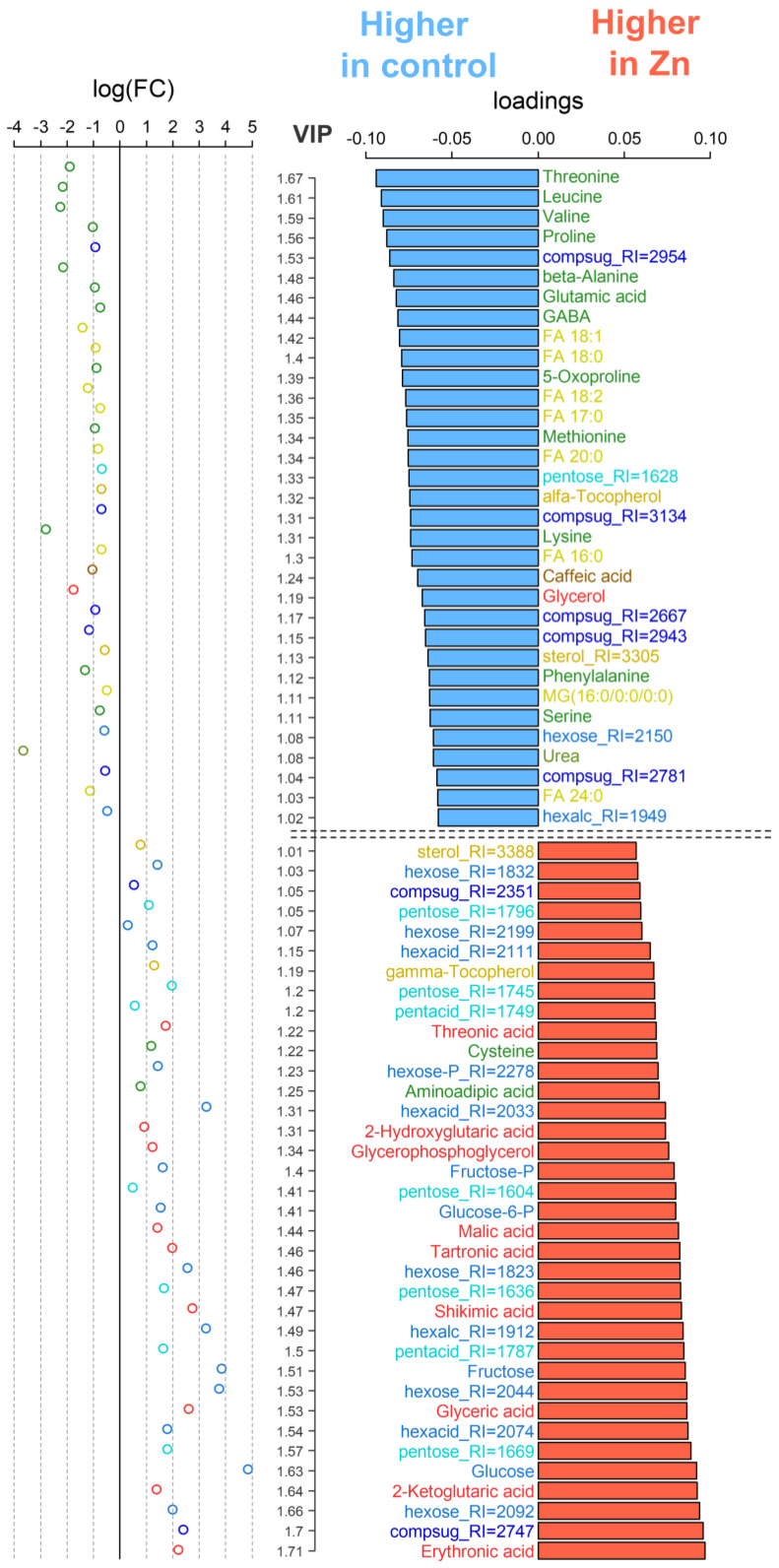
Selection of differentially accumulated metabolites (DAMs) in control and Zn-treated (Zn) plants. Factor loadings and the Variable Importance in Projection (VIP > 1, axis at the middle) from OPLS-DA model. Red bars and positive loadings correspond to increased accumulation in Zn-treated plants and vice versa. Scatter plots on the right are logarithmic ratios (FC, fold changes) of normalized content: log_2_(C_Zn_/C_control_). Colors of the bar labels mark chemical classes in a manner similar to Figure 2.

Next, OPLS-DA comparison between control and Zn-treated plants was performed. The model included a predictive component as well as a single orthogonal component. The predictive component, which is related to the effect of the factor of interest, explained 30% of the total variance. In this case, Q2Ypred = 0.81 (*p* = 0.02). Consequently, the observed differences in metabolite profiles between the control and +Zn groups are statistically significant and well-established. The loadings (VIP > 1) of the predictive component (Figure 4) clearly demonstrated that a total of 40 metabolites were found to be responsive to Zn excess.

Most metabolites had decreased contents (down-regulated), including fatty acids (FAs 17:0, 18:0; 18:1, and 18:2), amino acids (threonine, leucine, valine, proline, β-alanine, glutamic acid, 5-oxoproline, methionine, and serine), carboxylates (caffeic acid), and alcohols (glycerol), in comparison to the untreated control. The down-regulated free amino acids include the pivotal players in nitrogen metabolism, glutamic acid and GABA, which play a role in a wide range of metabolic pathways and are involved in the response to a variety of stimuli.

Among Zn-responsive metabolites, some metabolites were significantly increased (up-regulated) by Zn toxicity, such as carbohydrates, amino acids (cysteine), and carboxylates, including metabolites associated with the TCA cycle, such as 2-ketoglutarate and malate. Furthermore, this set encompasses glycerate, hydroxyglutarate, threonate, and erythronate. A key element in the synthesis of aromatic amino acids and many secondary compounds, shikimate, is also included in this range. Cysteine levels are higher in plants growing in the presence of Zn. This is probably due to the fact that this amino acid is involved in chelating heavy metal ions. Furthermore, Zn is also capable of reducing the concentration of free fatty acids (FFAs), although its effects on sterols are minimal. Zinc stimulates the accumulation of monosaccharides, including glucose and fructose, and their phosphates. In contrast, the complex sugars exhibited a multidirectional change, with some exhibiting a trend towards lower levels when Zn was added in excess.

**Figure 5:**
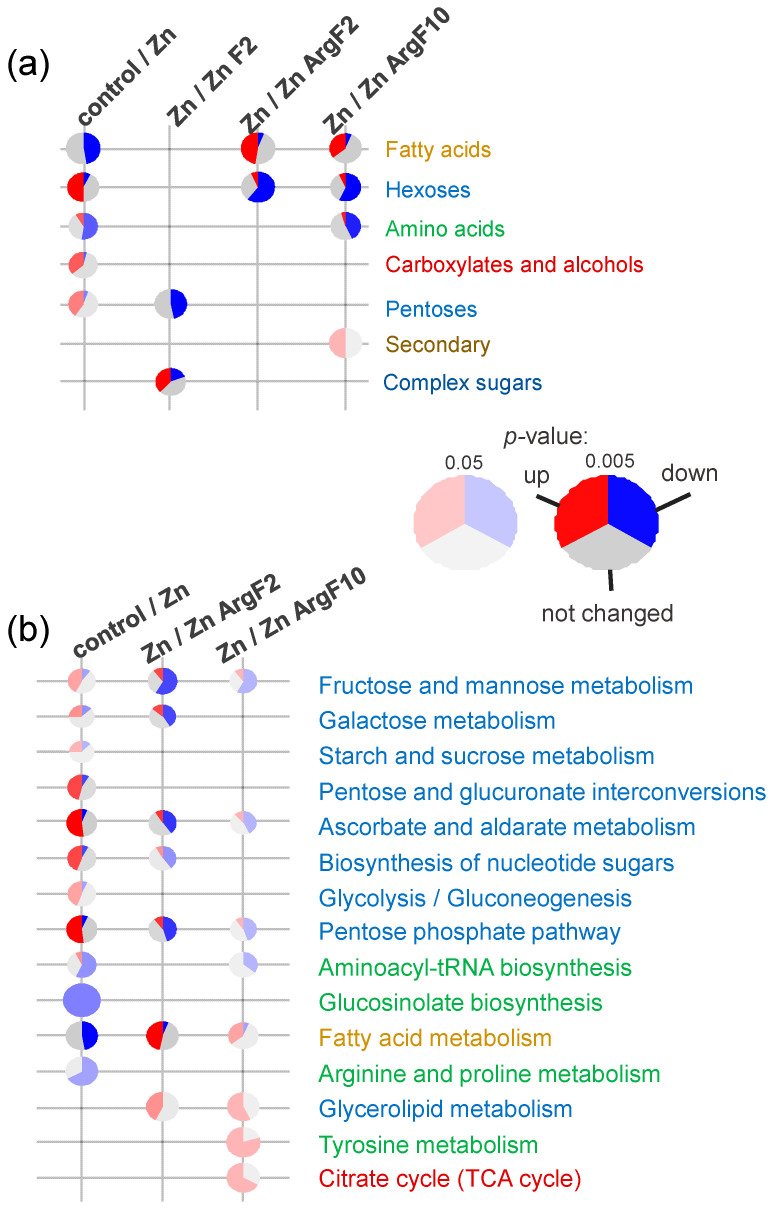
Metabolite set enrichment analysis based on chemical sets (**a**) and KEGG pathways (**b**). Pies represent proportions of differently accumulated metabolites (VIP > 1), which are up- or down-regulated in +Zn relative to control or in Zn + F or Zn + ArgF relative to Zn. The colors of metabolic pathways are derived by mixing the colors of the metabolites involved in the pathway according to the colors mentioned in Figure 2.

Metabolite set enrichment analysis (MSEA) resolved metabolically focused Zn effects (Figure 5a).

MSEA suggested that Zn stimulates the accumulation of monosaccharides. This may be due to an increase in the activity of pathways involved in carbohydrate metabolism (Figure 5b), such as the pentose phosphate pathway, pentose and glucuronate interconversions, fructose and mannose metabolism, glycolysis/gluconeogenesis, and others. Zinc also induced a greater accumulation of carboxylic acids, but this was not specifically related to the TCA cycle. Concurrently, Zn addition resulted in a decline in amino acid levels, which appears to be associated with the inhibition of arginine and proline metabolism and protein synthesis. Furthermore, the accumulation of FFA was also diminished.

**Figure 6:**
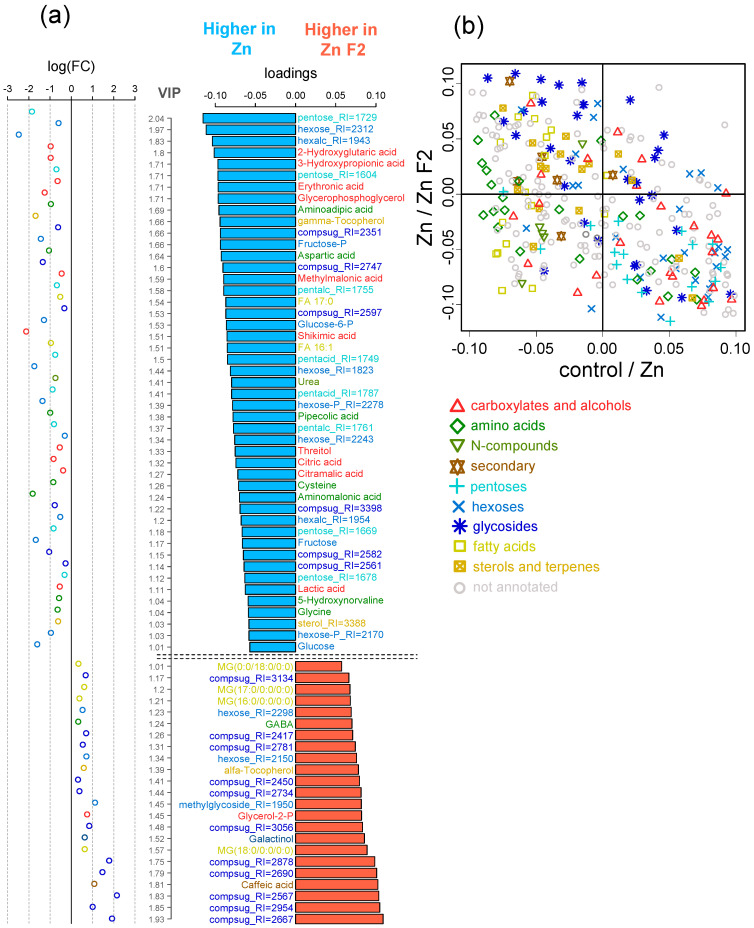
Effect of fullerenols on the metabolite profiles of the plants growing in the presence of Zn. (**a**) Selection of differentially accumulated metabolites (DAMs) in plants treated with Zn (Zn) and Zn and fullerenol with a concentration of 2 mg L^−1^ (Zn F2). Factor loadings and the Variable Importance in Projection (VIP > 1, axis at the middle) from OPLS-DA model. Red bars and positive loadings correspond to increased accumulation in Zn F2 plants and vice versa. Scatter plots on the right are logarithmic ratios (FC, fold changes) of normalized content: log_2_(C_Zn F2_/C_Zn_). Colors of the bar labels mark chemical classes in a manner similar to Figure 2. (**b**) SUS plot, scattering of metabolites in the space of loadings of predictive components from OPLS-DA models for comparison of the effect of excess Zn (abscissa) and the effect of F2 on plants in the presence of excess Zn (ordinate).

As mentioned above, the addition of fullerenols when Zn was present leads to some mild changes in the metabolic phenotype. To gain further insight, an OPLS-DA classification was performed. A more reliable model was revealed for Zn/ZnF2. The model included one orthogonal component, with 21% of the variance associated with the predictive component, Q2Ypred = 0.62 (*p* = 0.02). Five metabolites were increased in response to fullerenol (2) including complex sugars (galactinol), α-tocopherol, and caffeic acid (Figure 6a).

Conversely, fullerenol suppresses the accumulation of a wide range of compounds. These include numerous carboxylates, such as citrate and shikimate. Fullerenol also reduces pools of nitrogen-containing compounds, including aspartate, urea, cysteine, and glycine. A wide range of sugars, including monosaccharides such as glucose, fructose phosphate, and numerous pentoses, exhibited a reduction in response to fullerenol. However, the repressive effect on lipophilic compounds was relatively weak, but a decrease in the contents of γ-tocopherol and two fatty acids should be noted. Among the metabolites, there were some metabolites specifically involved in the Zn-stress responses of plants: glycerophosphoglycerol, erythronic acid, tocopherol, glucose-6-P, shikimic acid, cysteine, and caffeic acid. Changes in the contents of these metabolites caused by fullerenol and Zn treatments were opposite. The MSEA results (Figure 5) indicate that Zn exerts a repressive effect on monosaccharide metabolism and a stimulatory effect on the accumulation of complex sugars and lipid metabolism.

To elucidate the correlation between the effects of Zn and fullerenol, we conducted a comparative analysis of the OPLS-DA loadings of both the control/Zn and the Zn/ZnF2 models. The SUS plot (Figure 6b) demonstrated the opposite action of Zn and fullerenol, with a rho = −0.49 (Spearman’s correlation), indicating that the effects of Zn are significantly attenuated by fullerenes. This suggests that fullerenes moderately attenuate the effects of excessive Zn.

### 2.5. Metabolic Responses to Arginine Fullerene C_60_

**Figure 7:**
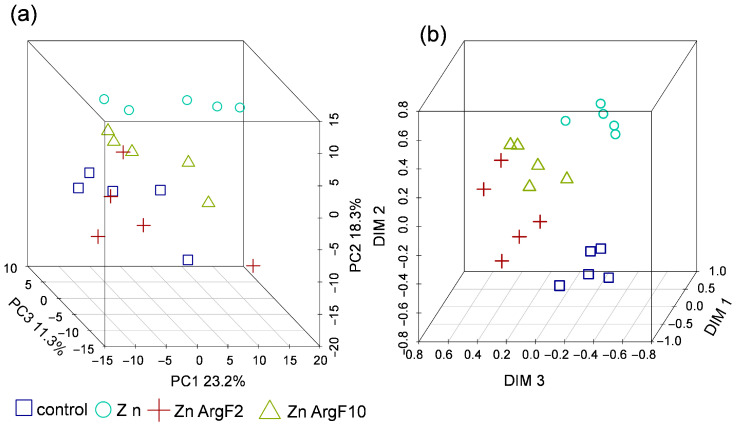
Metabolomic effects of excess Zn and arginine C_60_ derivative. (**a**) Score plots obtained by PCA. (**b**) Coordinates extracted by MDS using 1 rho as a distance measure. Dots correspond to the metabolite profiles, % is the percentage of variance related to the PC, and ellipses are 90% CI.

The metabolite profiles of the Zn + ArgF plants are shifted towards those of the control when plotted in a low-dimensional space (Figure 7).

**Figure 8:**
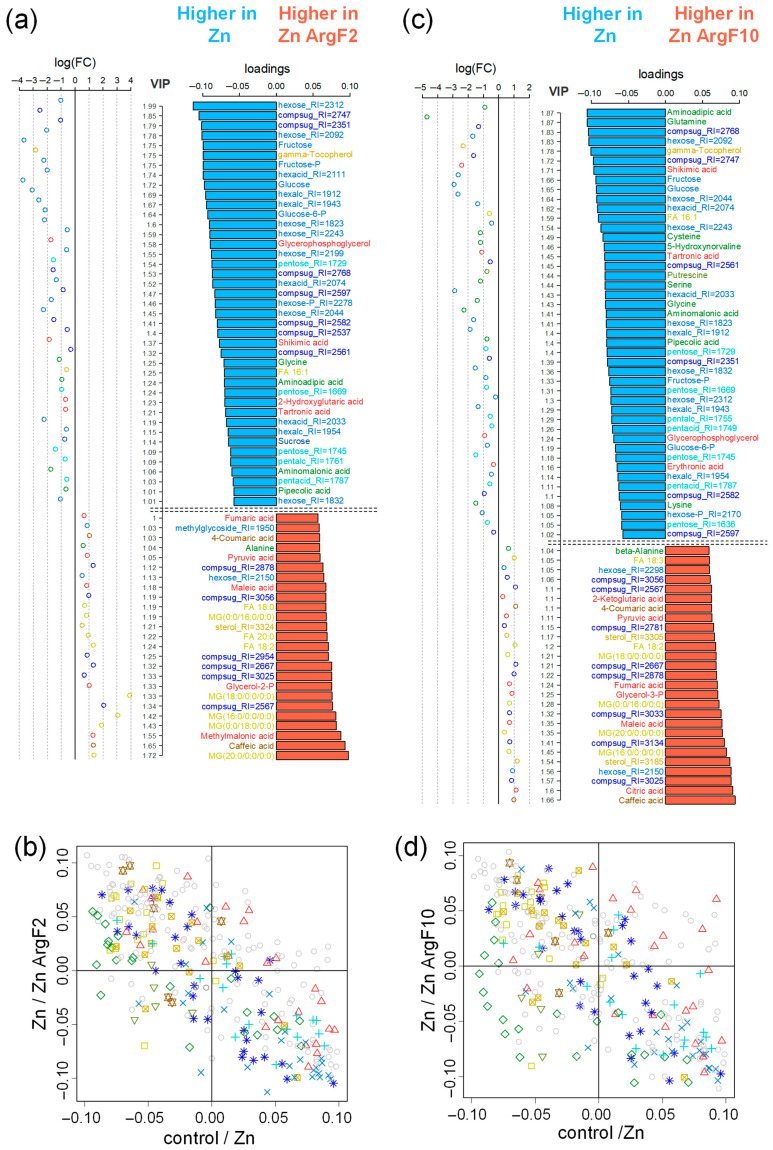
Metabolomic effects of arginine C_60_ derivative (ArgF). Selection of differentially accumulated metabolites (DAMs) in plants treated with Zn (Zn) and Zn and ArgF in two concentrations: (**a**) 2 mg L^−1^ (Zn ArgF2) and (**c**) 10 mg L^−1^ (Zn ArgF10). Colors of the bar labels mark chemical classes in a manner similar to Figure 2. Comparison of metabolomic effects of Zn and ArgF2 (**b**) or ArgF10 (**d**). SUS plots: scattering of metabolites in the spaces of loadings of predictive components from OPLS-DA models for comparison of the effects of Zn excess (abscissa) and the effect of ArgF on plants in the presence of excessive Zn (ordinate). The symbols are the same as in Figure 6b.

The effect of the ZnArgF2 is stronger, and such plants are indistinguishable from the control plants. OPLS-DA models were constructed to detect DAMs under the ArgF addition. The models included an orthogonal component, Q2Ypred = 0.65 (*p* = 0.04) and 0.7 (*p* = 0.04), and 24 and 22% of the variance were related to the predictive component for the Zn/ZnArg2 and Zn/ZnArg10, respectively. VIPs, loadings, and FDR are summarized (Tables S3 and S4). Bar plots of the loading (VIP > 1) (Figure 8a,c) demonstrate that some metabolites (about 15) exhibited down-regulation, while others (about 10) were up-regulated by the ArgF, depending on its dose. Among the metabolites, there were metabolites having opposite changes in their endogenous content in response to the ArgF and Zn.

For example, while an excess of Zn up-regulated hexose, pentose, glucose-6-P, glycerophosphoglycerol, tartronic, and shikimic acids, the ArgF2 down-regulated these metabolites in Zn-stressed plants. At the same time, the metabolites such as caffeic acid, diterbutylphenol, sterol, alanine, and fatty acids (18:0, 18:2, and 20:0) were increased by the ArgF2 and decreased by excessive Zn. After treatment with 10 µM ArgF, similar differences were observed for the ArgF2. The levels of shicimic, tartronic, and erytronic acids, as well as of cysteine, glycerophosphoglycerol, and glucose-6-P significantly decreased in the + ArgF10 compared to those in Zn-treated plants. In both cases, arginine C_60_ stimulates the accumulation of lipid metabolism compounds, especially monoacylglycerols, as well as some FFAs, which were suppressed by Zn, as was mentioned above. Another common feature of both variants is the suppression of the accumulation of monosaccharides, the level of which increases during Zn treatment, as was demonstrated earlier. Also, the ArgF suppresses the accumulation of some carboxylates and amino acids, which rises in response to Zn.

Nevertheless, it should be noted that other carboxylic acids such as pyruvate, methylmalonate, and maleate accumulated in large amounts in the ZnArgF2, while after Zn treatment, the levels of these metabolites were not significantly changed. We can also note a decrease in the level of aminoadipate, one of the few amino acids whose accumulation was induced by high Zn. A significant difference in the effect of arginine for the two concentrations is the effect on the carboxylate and amino acid profiles. The ArgF10 does not only suppress but even enhances the accumulation of some carboxylates; however, most of them were not involved in the response to Zn. The ArgF10 also represses the accumulation of a number of amino acids. However, an analysis of their composition indicates that these are not the amino acids that are primarily repressed by Zn. Despite the differences, the effects of arginine at two concentrations were similar.

The general effect of arginine was (1) to increase the accumulation of FFAs and their derivatives, as shown by the MSEA; (2) to inhibit monosaccharide metabolism, including PPP and the metabolism of fructose and mannose (Figure 5). The ArgF10 plants were also characterized by a reduced accumulation of amino acids against a background of increased TCA pools and tyrosine metabolism.

The comparative analysis of the effect of arginine fullerene C_60_ on Zn-treated plants (Figure 8b,d) shows that the effect of arginine C_60_ was generally opposite to that of Zn on the control plants. Moreover, the alleviating effect of ArgF2 (rho = −0.71) appears slightly stronger than that of ArgF10 (rho = −0.62). Additionally, a comparison of the effects of arginine C_60_ and fullerenol (2 mg L^−1^) showed their moderate similarity.

### 2.6. Removal Efficacy of Zn

Addition of the fullerene derivatives in an NS used for the hydroponic growth of cucumber did not affect the contents of water-soluble Zn added in an excessive amount: 15 and 40 µM (Table 2). The removal efficacy of Zn did not change with the increasing adsorbent dosage, at least not in concentration range from 2 to 10 mg L^−1^, indicating that, with an increase in available adsorption sites, the sorption capacity of fullerenes with respect to Zn remained low.

## 3. Discussion

In this study, retarded growth, losses of chlorophyll, and elemental imbalance were the typical symptoms of Zn-stress damage in cucumber plants (Figure 1 and Table 1). It has been reported that the inhibition of growth by excessive Zn is a result of limiting cell division, impaired cell elongation [30,31], and a drop in the intensity of physiology processes, including photosynthesis, water regime, and mineral nutrition [9,32]. Addition of fullerene C_60_ derivatives can partly alleviate Zn damage, mainly by suppressing the loss in the root dry biomass of Zn-stressed cucumber (Figure 1).

Here, we used a metabolomics approach to better understand the mechanisms of the alleviating effects of fullerene C_60_ derivatives in cucumber plants subjected to Zn excess. Resulting metabolite profiles showed that the control plants and Zn-stressed plants were different (Figure 3). After exposure to 40 µM Zn, the relative abundance of a large number of metabolites decreased in the leaves of cucumber compared to the control condition (Figure 4). On the one hand, the Zn toxicity appears to suppress arginine and proline metabolism and especially fatty acid metabolism (Figure 5). On the other hand, an excess of Zn increased the leaf abundance of nonstructural carbohydrates, carboxylates, and the amino acid cysteine, indicating that these metabolites might be involved in the adaptive responses of plants to abiotic stresses.

In plants, primary metabolites play essential roles in growth, development, and reproduction. Sugars are involved in most metabolic and signaling pathways that control growth, development, and stress tolerance in plants [33]. Carbohydrates are known to be involved in different stress responses, including heavy metal and oxidative stress [34]. In Zn-stressed amaranth, most of the responsive metabolites were up-regulated and dominated with sugars [35].

Organic acids such as malate and 2-ketoglutarate are involved in the TCA cycle, being the major energy production pathway. In addition, organic acids can act as efficient chelating agents for HMs. Organic acids played key roles in the regulation of metabolism in Zn-stressed tea leaves [36]. Whereas Zn toxicity leads to drastic depression in the N metabolism, the abundance of cysteine in cucumber leaves increased. Cysteine is a reduced sulfur donor molecule that has a central role in plant metabolism. Cysteine-based redox regulation and signaling are essential for plant responses to different stress conditions [37,38]. Changes in tocopherol levels contribute to plant tolerance against oxidative stress. It is generally assumed that increases in α-tocopherol contribute to plant stress tolerance, while decreased levels favor oxidative damage [39]. It is thought that secondary metabolites, including phenolic compounds, reflect plant environments better than primary metabolism. Shikimates play important roles in the defense mechanisms of plants, acting as pigments, antioxidants, and signaling agents [40].

Our results showed that adducts of fullerene C_60_ can change the physiological and metabolic responses of cucumber plants to Zn excess in a dose-dependent manner, being overall more pronounced at a concentration of 2 mg L^−1^. Generally, both fullerenol and ArgF affected the total metabolism of Zn-stressed cucumber. Fullerenol repressed the metabolism of monosaccharides and stimulated the production of complex sugars (Figure 5 and Figure 6), while ArgF activated the fatty acid metabolism and depressed the pentose phosphate pathway, ascorbate and aldarate metabolism, and fructose and mannose metabolism (Figure 5 and Figure 8).

We identified metabolites not-specifically and specifically involved in Zn-stress responses. Galactinol, glycerol-2-P, α-tocopherol, and glycerophospholipids were fullerenol-increased metabolites, which relative abundance did not significantly alter when plants grew under excessive Zn conditions without fullerene adducts (Figure 6). It is well known that raffinose and its precursor, galactinol, accumulate in plant leaves during abiotic stress [41]. Plant drought tolerance was enhanced through either raffinose synthesis or galactinol hydrolysis, depending on sucrose availability in plant cells [42]. Furthermore, both galactinol and α-tocopherol scavenge hydroxyl radicals to protect plant cells from oxidative damage caused by different abiotic stress conditions [39]. Enhancing lipid-like molecules (glycerophospholipids) in leaves positively correlates with the stress tolerance of plants [43].

Interestingly, aspartic acid was among those metabolites that were down-regulated by fullerenol treatments (Figure 6), although abundance of this acid was not altered by the excessive addition of Zn. The organic acid is of importance to the biosynthesis of other amino acids, organic acids, and sugars in glycolysis [44]. Under stress conditions, the endogenous content of aspartate may increase or decrease according to plant species and the type of stress [44]. Aspartate is thought to play a role in modulating plant tolerance against biotic and abiotic stresses. Taken together, the increased content of galactinol and α-tocopherol against a background of decreased aspartate in the leaves of fullerenol-treated plants suggest that fullerenol offers advantages in overcoming oxidative damage caused by Zn excess.

In arginine-treated plants, we also found an increased abundance of glycerol-2-P and, additionally, some carboxylates, including pyruvate (Figure 8). Pyruvate plays a key role in the metabolism of sugar. Moreover, it is an intermediate in several metabolic pathways closely associated with nitrogen and fat metabolism through the acetyl-CoA and TCA cycles [45]. Since these cycles are involved in producing energy sources such as ATP and NADH, we speculate that arginine C_60_ might activate energy consumption in the leaves of Zn-stressed plants.

In addition, fullerene adducts altered the leaf abundance of some Zn-responsible metabolites in an opposite direction when compared with effects of high Zn on the control plants (Figure 6 and Figure 8). Whereas high Zn down-regulated some metabolites, fullerene treatment up-regulated their content and vice versa. Among fullerenol-altered Zn-responsible metabolites, we found carbohydrates, glucose-6-P, γ-tocopherol, shikimic acid, erythronic acid, glycerophosphoglycerol, cysteine, and caffeic acid. The list of such metabolites affected by ArgF included carbohydrates, glucose-6-P, shikimic acid, tartronic acid, glycerophosphoglycerol, sterol, fatty acids (FA 18:0, 18:2, and 20:0) alanine, diterbutylphenol, and caffeic acid. The relative abundance of some metabolites was altered similarly by both fullerene C_60_ treatments. Indeed, with respect to the Zn-suffering plants, both derivatives down-regulated the abundance of some carbohydrates, glycerophosphoglycerol, glucose-6-P, and shikimic acid, and they up-regulated caffeic acid. Since the fullerene-induced alterations were in opposition to the Zn treatments’ directions, the results indicate that derivatives of C_60_ might at least partly alleviate Zn-depressed plant growth by accumulating some primary and secondary metabolites.

It is well known that heavy metal accumulation can initiate oxidative stress by generating ROS [9,46]. Nanoparticles can ameliorate HM toxicity in plants. On the one hand, ENMs (e.g., TiO_2_) have the ability to reduce oxidative stress and alleviate HM toxicity through signaling that triggers antioxidant enzyme activities in stressed plants [15]. On the other hand, ENMs could limit the translocation from roots to shoots due to adsorption effects exerted by ENMs [15].

Although the exact mechanisms of fullerene-induced mutual influences are not yet clear, many authors have been reported that fullerenols can protect crops against oxidative stress induced by different stressful environments [19,22,23]. It has been hypothesized that fullerenol can act as a scavenger of free radicals [19,23]. In this study, we did not determine the activity of antioxidant enzymes and ROS. However, we found that fullerene derivatives increased the abundance of some metabolites (galactinol and α-tocopherol) involved in antioxidant defense mechanisms. Both galactinol and α-tocopherol scavenge hydroxyl radicals to protect plant cells from oxidative damage caused by different abiotic stress conditions [39].

Recently, we demonstrated that fullerenol, and especially arginine-functionalized fullerene C_60_, effectively alleviated symptoms of Cu toxicity due to Cu buffering in the root zone, causing a decline in the Cu transport from roots to leaves [26]. Contrastingly, both fullerenol and arginine C_60_ exhibited a relatively low Zn-uptake adsorption capacity and therefore did not restrict excessive Zn translocation towards cucumber leaves. Many authors have demonstrated that the interactions of metal ions with appendant groups of functionalized fullerenes could lead to corresponding coordination complexes [47]. It has been reported that many obtained fullerenol complexes chelated with heavy metal ions, including Zn^2+^, were insoluble [48,49]. Complexation of fullerenols with Cu^2+^ consists of two processes: electrostatic interaction of Cu^2+^ with negatively charged oxygen-contained groups of fullerenol and then the coordination of Cu as Cu-O bonds. Observed alleviation of Cu-toxicity in maize could be due to fullerenol partially converting free Cu^2+^ into the binding state of Cu^2+^-nanocomposites, leading to a decrease in Cu content in plants and the content of free Cu^2+^ ions in vivo [28]. Contrastingly, the formation of insoluble Zn compounds after adding fullerene derivatives was not observed, indicating that they were not involved in the removal of Zn ions from contaminated nutrient solutions by simply mixing in the different molar ratios (Table 2). Taken together, the results indicate that fullerenol and arginine C_60_ exhibited potentially protective effects on cucumber plants and diminished the toxic effects induced by Zn excess. As a whole, the ameliorative effects of C_60_ derivatives on Zn-stressed cucumber were relatively weak. That was because the examined adducts of C_60_ exhibited a low sorption capacity and thereby a low removal efficacy of Zn under the conditions of our experiments.

## 4. Materials and Methods

### 4.1. Characterization of Fullerene C_60_ Derivatives

In this paper, fullerenol [C_60_(OH)_22–24_] and the C_60_ fullerenes adduct with L-arginine [C_60_(C_6_H_13_N_4_O_2_)_8_H_8_] were under study. These derivatives were obtained by methods of Semenov et al. [50] and Hu et al. [51], respectively. In our earlier papers [25,26,27], details of the synthesis and identification methods for these C_60_ derivatives were described. We used a group of physicochemical methods to identify the fullerene C60 derivatives: IR spectroscopy (Shimadzu FTIR-8400S spectrometer, Japan), elemental analysis (EuroVector Euro EA3028-HT, Italy), mass spectrometry (Shimadzu MALDI-TOF mass spectrometer Axima–Resonance, Japan), UV spectroscopy (Shimadzu UV-1800 spectrophotometer, Japan), 13C NMR spectroscopy (NMR spectrometer Bruker Avance III 400 WB, San Jose, CA, USA), and complex thermal analysis (Shimadzu DTG-60H). The physical and chemical properties of these derivatives (hydrodynamic diameter and zeta potential) as well as of their associations with Zn were detected as described previously [26]. Some materials’ properties are shown in Table A2.

### 4.2. Plant Materials and Growth

Seeds of cucumber (*Cucumis sativus* L., *cv*. Solovey) were obtained from the Vavilov Research Institute plant genetic resources (Saint Petersburg, Russia). The seeds were germinated in the dark at 28 °C for 4 days. Over the next 7 days, the seedlings were pre-incubated without excessive Zn in a complete nutrient solution (NS) containing the following: (mM): 0.7 K_2_SO_4_, 0.1 KCl, 2.0 Ca(NO_3_)_2_ 4 H_2_O, 0.5 MgSO_4_ 7·H_2_O, and 0.1 KH_2_PO_4_ and (µM): 1.0 MnSO_4_, 1.0 ZnSO_4_, 0.5 CuSO_4_·5H_2_O, 0.01 (NH_4_)_6_Mo_7_O_24_, 10 H_3_BO_3_, and 10 Fe(III)–EDTA. The plants were grown in 1 L plastic pots (two plants per pot). 

Then, the seedlings were grown for 1–17 days in the basal solutions with six compositions: (1) basic NS with 1.0 µM Zn (control), (2) with 40 µM Zn, (3) with 40 µM Zn and 2 mg L^−1^ fullerenol (F2), (4) with 40 µM Zn and 10 mg L^−1^ fullerenol (F10), (5) with 40 µM Zn and 2 mg L^−1^ arginine C_60_ (ArgF2), and (6) with 40 µM Zn and 10 mg L^−1^ arginine C_60_ (ArgF10). Zinc was applied as Zn sulfate at 40 µM. The concentration that was moderately toxic for cucumber was estimated based on a preliminary screening experiment with 1, 40, and 80 µM Zn. Recently, we found that the fullerene concentrations used were effective in experiments with Cu toxicity in cucumber [26]. The pH was adjusted to 6.0. The photoperiod was 16 h light/8 h dark. Irrigation was 200 µmol m^−2^ s^−1^ proton flux density provided by fluorescent lamps; relative humidity of the air—50–60%.

### 4.3. Physiological Evaluation of Zn Toxicity

During the growing period, leaf chlorophyll (Chl) content was determined using a portable Chl meter (SPAD-502, Minolta Camera Co., Osaka, Japan) and was presented as SPAD (Spectral Plant Analyses Diagnostic) index values. Measurements were carried out in different leaves from the base to the youngest leaf. Data were collected from two leaves in each pot. At harvest, plants were divided into roots, stems (together with leaf petioles), and leave (blades) and dried at 70 °C for 48 h. Dry weight was used as a measure of growth.

Xylem sap collection was carried out after the stems were cut 2 cm above the roots. We collected xylem sap with a micropipette for 1 h. In freshly collected xylem sap, Zn concentration was detected by electrothermal atomic absorption spectrometry (ETAAS; MGA 915, Lumex, Russia).

The concentrations of macro- and microelements in cucumber leaves and roots were detected by inductively coupled plasma optical emission spectroscopy (Shimadzu ICPE-9000, Shimadzu Corp., Japan). Oven-dried samples of plants (0.1 g) were microwave-digested (Minotavr-2, Lumex, Russia; MDS-10, Sineo Microwave Chemistry Technology Co., Ltd., China) in concentrated HNO_3_. Then, samples were placed into 10 mL volumetric flasks. The standard samples of the analyzed elements for calibration solutions were prepared from multicomponent and monocomponent standards (Merck, Germany) in 0.1 M HNO3. Range of calibration solutions: 0.001–100 mg L^−1^. The spectral analysis of the sample solution was carried out in the axial view configuration without dilution and with a dilution of 100 times. The emission intensities were measured at the most sensitive spectral lines (mn): Ca (183.801), Cu (324.754), Fe (259.940), K (793.867), Mg (383.826), Mn (260.569), P (177.499), S (180.731), Zn (206.200), and B (249.678).

### 4.4. Gas Chromatography–Mass Spectrometry (GC-MS) of the Leaves

The procedures of metabolite extraction, derivatization, and analysis of extracts using GC-MS were described previously [25,27]. Tricosane, linear C_23_ alkane, was used as internal standard. Mixture of linear alkanes was used for retention index (RI) calibration. GC-MS analysis was conducted using an Agilent 6850 chromatograph coupled with an Agilent 5975 mass selective detector under control of the Agilent MassHunter B 07.05 software (Agilent Technologies, Santa Clara, CA, USA). For chromatographic separation, the Rxi-Sil-5MS capillary column was employed (Restek, Bellefonte, PA, USA). The following setup was applied: the inlet temperature was 250 °C, the splitless mode was employed, the helium flow rate was 1 mL min^−1^, the initial temperature was set at 70 °C, and the rate of growth was 6 °C per min up to 320 °C.

### 4.5. GC-MS Data Interpretation and Statistical Analysis

The procedure has been described previously [27]. Briefly, processing of the data files was conducted in PARADISe 6.01 software [52], coupled with NIST MS Search 2.4 (National Institute of Standards and Technology, USA). For the purpose of additional metabolite identification, the AMDIS 2.71 (Automated Mass Spectral Deconvolution and Identification System; NIST, USA) was utilized. The identification of the mass spectra was conducted by comparing them with records in the NIST2020, Golm Metabolome Database (GMD), and in-house libraries of the Laboratory of Analytical Phytochemistry BIN RAS (assignment # 124020100140-7) and the Resource Center “The Development of Molecular and Cell Technologies” of the St. Petersburg University. A mass spectrum was considered reliably identified if it corresponded to the library entry, exhibiting a matching factor of at least 800. Secondly, retention indices (RIs) were determined by calibration with standard alkanes (C_10_–C_40_) and subsequently employed in the process of identification. For some peaks that could not be accurately identified, the chemical class was annotated.

Data was processed in the environment of the R language 4.3.1 [53]. The data were normalized by sample median, log-transformed, and standardized. If a metabolite was not detected but was present in other replicate samples, it was postulated to be a technical error, and values were imputed using KNN (k-nearest neighbors) in the *impute* package [54]. Principal component analysis (PCA) was made using the *pcaMethods* [55]. OPLS-DA models were developed with *roopls* package [56]. The heatmap was generated using the *ComplexHeatmap* package [57]. The loadings of the predictive components from OPLS-DA models were employed as a ranking statistics factor for metabolite set enrichment analysis (MSEA). The U-test was applied for MSEA using the *tmod* package [58]. Metabolite sets for metabolite pathways for *M. truncatula* were downloaded from the KEGG database [59] using *KEGGREST* 1.51.1 [60].

Four–five replicate pots were used per treatment. The analyses of variance (Type III ANOVA: IBM SPSS Statistics, version 27) was studied for the data (biomass, leaf Chl, and elemental content), and the mean differences between treatments were tested at 5% significance level (*p* < 0.05) using Student–Newman–Keul’s test.

## 5. Conclusions

The Zn-treated plants show chlorotic leaves, retarded growth of shoots (−20%) and roots (−49%), and nutrient imbalance. Addition of fullerene C_60_ derivatives suppressed loss in the dry biomass of leaves (15%) and roots (40%; fullerenol only) induced by high Zn. However, they did not alter leaf chlorophyll, shoot dry biomass, and elemental composition including leaf Zn and xylem sap Zn. Metabolite profiling in stressed plants was slightly altered in the presence of C_60_ derivatives. However, in fullerene-treated plants, the abundance of some Zn-responsible metabolites tended to change in the opposite direction in comparison to the metabolic responses to excessive Zn alone. Among the up-regulated metabolites, there were those (e.g., galactinol and α-tocopherol) that are involved in antioxidant defense mechanisms. We speculate that fullerene C_60_ derivatives could alleviate Zn toxicity by the stimulation of antioxidant non-enzyme activity at least. However, these alterations in plant defense mechanisms were not enough to reduce the transport of Zn excess from the roots to shoots. Fullerenes by themselves did not buffer Zn in the root zone. We conclude that the relatively low tolerance of Zn-stressed cucumber in the presence of C_60_ derivatives was related to their surface chemistry, which caused the selectively low removal efficacy of Zn in the root zone of plants. The results provide new evidence for the crucial role of the Zn-sorption capability of fullerene adducts C_60_ in the Zn tolerance of cucumber plants.

## Figures and Tables

**Table 1:** The effect of excess Zn on contents of micro- and macronutrients in leaves of cucumber grown in a nutrient solution with or without the supply of 2 (F2) and 10 (F10) mg L^−1^ fullerenol or 2 (ArgF2) and 10 (ArgF10) mg L^−1^ arginine C_60_ for 17 d. Data represented as mean ± SD (*n* = 4). Significant differences between treatments (*p* < 0.05) are indicated by different letters.

Treatments	B	Fe	Zn	Mn	Cu	P	K	S	Ca	Mg
	μg Plant^−1^ DW					mg Plant^−1^ DW				
Control	48 ± 4 b	104 ± 10 b	44 ± 4 a	56 ± 7 a	12 ± 1 a	9.5 ± 0.5 c	47 ± 2 b	6.1 ± 0.4 c	98 ± 8 b	4.2 ± 0.5 a
Zn	35 ± 3 a	54 ± 8 a	542 ± 31 b	48 ± 2 a	10 ± 2 a	5.7 ± 0.6 a	37 ± 3 a	4.7 ± 0.6 a	70 ± 7 a	3.3 ± 0.5 a
Zn F2	41 ± 5 ab	63 ± 8 a	579 ± 79 b	53 ± 6 a	11 ± 1 a	6.8 ± 0.5 ab	40 ± 3 a	5.8 ± 0.3 bc	79 ± 1 ab	3.8 ± 0.3 a
Zn F10	32 ± 5 a	54 ± 12 a	543 ± 42 b	48 ± 3 a	9 ± 1 a	6.7 ± 1.3 ab	36 ± 1 a	5.1 ± 0.6 ab	75 ± 10 a	3.5 ± 0.4 a
Zn ArgF2	41 ± 6 ab	52 ± 9 a	635 ± 112 b	54 ± 3 a	10 ± 2 a	7.0 ± 0.9 ab	40 ± 3 a	5.3 ± 0.4 abc	85 ± 14 ab	4.0 ± 0.4 a
Zn ArgF10	41 ± 4 ab	52 ± 5 a	649 ± 82 b	54 ± 3 a	9 ± 1 a	7.5 ± 0.5 b	36 ± 3 a	4.6 ± 0.4 a	78 ± 10 ab	3.8 ± 0.6 a

**Table 2:** Zinc concentrations in a nutrient solution (NS) after incubation for 1 d with or without the fullerene C_60_ derivatives at different molar ratios between Zn and fullerene derivatives (FD). Data represented as mean ± SD (*n* = 3). Significant differences between treatments (*p* < 0.05) are indicated by different letters.

Sample	Zn, μM	Molar Ratio C_Zn_:C_FD_
At 15 μM ZnSO_4_		
NS	1.0 ± 0.1 a	1:0
NS + 15 μM ZnSO_4_	14.2 ± 0.3 b	15:0
NS + 15 μM ZnSO_4_ + 2 mg L^−1^ C_60_(OH)_22–24_	14.4 ± 1.8 b	15:1.8
NS + 15 μM ZnSO_4_ + 10 mg L^−1^ C_60_(OH)_22–24_	15.3 ± 1.9 b	15:8.9
NS + 15 μM ZnSO_4_ + 2 mg L^−1^ C_60_ (Arg)_8_	15.8 ± 0.9 b	15:0.95
NS + 15 μM ZnSO_4_ + 10 mg L^−1^ C_60_ (Arg)_8_	14.5 ± 1.4 b	15:4.7
At 40 μM ZnSO_4_		
NS	0.97 ± 0 a	1:0
NS + 40 μM ZnSO_4_	38.8 ± 5 b	40:0
NS + 40 μM ZnSO_4_ + 2 mg L^−1^ C_60_(OH)_22–24_	37.8 ± 3 b	40:1.8
NS + 40 μM ZnSO_4_ + 10 mg L^−1^ C_60_(OH)_22–24_	38.8 ± 4 b	40:8.9
NS + 40 μM ZnSO_4_ + 2 mg L^−1^ C_60_ (Arg)_8_	41.7 ± 1 b	40:0.95
NS + 40 μM ZnSO_4_ + 10 mg L^−1^ C_60_ (Arg)_8_	42.7 ± 3 b	40:4.7

## Data Availability

The data that support this study are available in the article.

## Supplementary Materials

The following supporting information can be downloaded at: https://www.mdpi.com/article/10.3390/plants15020254/s1, Table S1. Differential metabolite normalized arbitary accumulation in control and Zn treated plants. Table S2. Differential metabolite normalized arbitary accumulation in Zn and ZnF2 treated plants. Table S3. Differential metabolite normalized arbitary accumulation in Zn and ZnArgF2 treated plants. Table S4. Differential metabolite normalized arbitary accumulation in Zn and ZnArgF10 treated plants.

## Appendix A

**Table A1:** The effect of excess Zn on contents of micro- and macronutrients in leaves and roots of cucumber grown in a nutrient solution with or without the supply of 2 (F2) and 10 (F10) mg L^−1^ fullerenol or 2 (ArgF2) and 10 (ArgF10) mg L^−1^ arginine C_60_ for 1 d. Data represented as mean ± SD (*n* = 4). Significant differences between treatments (*p* < 0.05) are indicated by different letters.

Treatments	B	Fe	Zn	Mn	Cu	P	K	S	Ca	Mg
	Leaves									
	μg plant^−1^ DW					mg plant^−1^ DW				
Control	1.6 ± 0.7 a	4.5 ± 1.1 a	3 ± 0.3 a	3.2 ± 0.5 a	0.38 ± 0.04 bc	0.39 ± 0.04 a	1.38 ± 0.21 a	0.22 ± 0.03 a	2.1 ± 0.4 a	0.16 ± 0.02
Zn	1.7 ± 0.9 a	3.3 ± 0.3 a	13 ± 1 b	2.5 ± 0.4 a	0.47 ± 0.16 b	0.41 ± 0.03 a	1.35 ± 0.13 a	0.23 ± 0.03 a	2.0 ± 0.4 a	0.14 ± 0.02
Zn F2	1.5 ± 0.5 a	4.2 ± 0.6 a	12 ± 2 b	2.5 ± 0.2 a	0.36 ± 0.13 bc	0.40 ± 0.04 a	1.38 ± 0.19 a	0.23 ± 0.03 a	2.0 ± 0.4 a	0.14 ± 0.04
Zn F10	1.3 ± 0.2 a	3.5 ± 0.9 a	12 ± 1 b	2.6 ± 0.7 a	0.33 ± 0.07 bc	0.40 ± 0.09 a	1.41 ± 0.23 a	0.22 ± 0.06 a	1.9 ± 0.4 a	0.14 ± 0.03
Zn ArgF2	1.4 ± 0.1 a	3.9 ± 0.5 a	15 ± 2 b	2.7 ± 0.4 a	0.26 ± 0.06 a	0.44 ± 0.09 a	1.42 ± 0.25 a	0.26 ± 0.05 a	2.3 ± 0.4 a	0.15 ± 0.02
Zn ArgF10	1.2 ± 0.3 a	3.4 ± 0.4 a	13 ± 2 b	2.5 ± 0.4 a	0.23 ± 0.13 a	0.41 ± 0.08 a	1.29 ± 0.22 a	0.23 ± 0.05 a	1.9 ± 0.5 a	0.13 ± 0.03
	Roots									
	μg plant^−1^ DW					mg plant^−1^ DW				
Control	0.10 ± 0.04 a	12 ± 1 a	1.9 ± 0.3 a	4.1 ± 0.4 b	1.0 ± 0.2 a	0.14 ± 0.02 a	1.0 ± 0.1 a	0.04 ± 0.01 a	0.10 ± 0.02 a	0.03 ± 0.01 a
Zn	0.15 ± 0.06 a	16 ± 6 a	16 ± 3 b	1.1 ± 0.2 a	2.2 ± 0.5 c	0.17 ± 0.04 a	1.4 ± 0.3 a	0.04 ± 0.01 a	0.12 ± 0.02 a	0.03 ± 0.01 a
Zn F2	0.16 ± 0.05 a	13 ± 2 a	17 ± 3 b	1.3 ± 0.4 a	2.1 ± 0.1 bc	0.16 ± 0.01 a	1.2 ± 0.2 a	0.04 ± 0.01 a	0.11 ± 0.01 a	0.03 ± 0.01 a
Zn F10	0.18 ± 0.05 a	11 ± 2 a	14 ± 2 b	1.2 ± 0.2 a	1.5 ± 0.2 ab	0.13 ± 0.03 a	1.1 ± 0.2 a	0.04 ± 0.01 a	0.10 ± 0.02 a	0.02 ± 0.01 a
Zn ArgF2	0.16 ± 0.05 a	14 ± 3 a	15 ± 2 b	1.3 ± 0.2 a	2.0 ± 0.4 bc	0.16 ± 0.03 a	1.3 ± 0.3 a	0.04 ± 0.01 a	0.11 ± 0.03 a	0.03 ± 0.01 a
Zn ArgF10	0.31 ± 0.06 b	15 ± 3 a	16 ± 2 b	1.3 ± 0.2 a	1.9 ± 0.3 bc	0.13 ± 0.02 a	1.0 ± 0.2 a	0.04 ± 0.005 a	0.09 ± 0.03 a	0.02 ± 0.01 a

**Table A2:** Hydrodynamic diameters of associations in binary (C_60_(OH)_22–24_–H_2_O; C_60_(Arg)_8_–H_2_O) and ternary(C_60_(OH)_22–24_–ZnSO_4_–H_2_O; C_60_(Arg)_8_–ZnSO_4_–H_2_O) systems at pH 5.5. The data are represented as median ± SD.

Sample	Hydrodynamic Diameter (nm)
10 mg L^−1^ C_60_(OH)_22–24_	60 ± 20
40 μM CuSO_4_ + 10 mg L^−1^C_60_(OH)_22–24_	91 ± 10
10 mg L^−1^ C_60_ (Arg)_8_	150 ± 20
40 μM ZnSO_4_ + 10 mg L^−1^ C_60_ (Arg)_8_	139 ± 3
